# Exposed Necrotic Bone in a Head and Neck Cancer Patient: Report of a Diagnostic Challenge

**DOI:** 10.3390/diagnostics15080952

**Published:** 2025-04-09

**Authors:** Fabio Dell’Olio, Rosaria Arianna Siciliani, Marta Forte, Saverio Capodiferro, Gianfranco Favia, Luisa Limongelli

**Affiliations:** Complex Operating Unit of Odontostomatology, Department of Interdisciplinary Medicine, Aldo Moro University, 70124 Bari, Italy; r.siciliani2@studenti.uniba.it (R.A.S.); fmarta@live.it (M.F.); saverio.capodiferro@uniba.it (S.C.); gianfranco.favia@uniba.it (G.F.); luisa.limongelli@uniba.it (L.L.)

**Keywords:** cutaneous squamous cell carcinoma, head and neck cancer, MRONJ, osteoradionecrosis, cemiplimab, denosumab, guidelines, radiology, surgical pathology, oral surgery

## Abstract

**Background and Clinical Significance:** The current study aims to show the diagnostic challenge of mandibular exposed necrotic bone in a patient with locally aggressive cutaneous squamous cell carcinoma of the lower lip and carrying risk factors for osteoradionecrosis and medication-related osteonecrosis of the jaws. **Case Presentation:** In March 2023, an 80-year-old ex-farmer male patient complaining of feeding difficulty showed a 3 cm area of exposed bone in the left region of the mandible. In July 2020, the patient underwent an incisional biopsy of a lower labial cutaneous keratinizing squamous cell carcinoma, which developed within actinic cheilitis. The cancer was unresectable due to the extent of the local invasion; thus, the patient underwent radiotherapy. In February 2022, the cancer reached the left mandibular canal by completely infiltrating the homolateral canal of the mental nerve. Therefore, the oncologist prescribed cemiplimab and denosumab as palliative immunotherapy. The differential diagnosis included osteoradionecrosis, stage-III medication-related osteonecrosis of the jaws, and intraoral localization of the cutaneous squamous cell carcinoma. The oral surgeon performed a sequestrectomy under local anesthesia and antibiotic prophylaxis; a histological examination confirmed the hypothesis of medication-related osteonecrosis. The patient currently undergoes follow-up visits monthly; the combination of photobiomodulation therapy and cycles of antibiotics keeps the necrotic lesion steady, and the oncological therapy prevents the growth of the cutaneous squamous cell cancer. **Conclusions:** The current case supports the need for histological examination to resolve the diagnostic challenge of mandibular exposed necrotic bone and to differentiate among osteoradionecrosis, stage-III medication-related osteonecrosis of the jaws, and intraoral localization of cutaneous squamous cell carcinoma.

## 1. Introduction

Exposed necrotic bone of the jaws (ENB) occurs in patients with disrupted bone homeostasis, causing bone destruction, loss of oral functions, and impairment of their quality of life (QoL) [1]. Indeed, patients with ENB report pain, halitosis, and dysgeusia as the most common symptoms; in addition, local or systemic infections, intraoral or extraoral fistulas, trismus, numb chin syndrome, and pathological fractures occur in advanced cases [2,3]. The medical history of patients showing ENB can involve single or multiple concurring potential etiologic agents, such as ionizing radiation therapy, anti-resorptive drug treatment, bone inflammation, and local or distant malignancies causing bone destruction [1]. However, the differential diagnosis of the primary disease causing ENB is challenging because of the overlapping clinical and radiological appearance of such clinical conditions, and several authors suggest that histological examination should be mandatory for all osteolytic lesions and that malignancy suspicion should always be high [2,4,5]. The current literature recognizes several diseases that can lead to ENB and require targeted treatment approaches, such as medication-related osteonecrosis of the jaw (MRONJ), osteoradionecrosis (ORN), and squamous cell carcinoma (SCC) [1,4,6,7,8]. MRONJ is an infective complication of anti-resorptive therapies and has a progressive course; therefore, the treatment involves antibiotics and surgical bone resection [9]. ORN needs conservative nonsurgical treatment and shows an indication for antibiotic therapy in cases of bacterial over-infection; only patients carrying large areas of exposed bone undergo surgery [10]. Cutaneous SCC requires surgery or radiotherapy in >95% of patients; the remaining <5% show metastatic or locally advanced cutaneous SCC, which is not eligible for surgery or radiotherapy and undergoes chemotherapy [11]. Therefore, differential diagnosis among the diseases causing ENB is pivotal for treatment and prognosis. The current guidelines of the American Association of Oral and Maxillofacial Surgeons (AAOMS) established the three diagnostic criteria for MRONJ [7]: first, current or previous treatment with antiresorptive therapy alone or in combination with immune modulators or antiangiogenic medications; second, exposed bone or bone that can be probed through an intraoral or extraoral fistula(e) in the maxillofacial region that has persisted for more than eight weeks; third, no history of radiation therapy to the jaws or metastatic disease in the jaws [7]. The radiographic findings of MRONJ include bone sequestrations, lytic changes, sclerosis, thickening of the lamina dura, and periosteal bone formation [4]. An ORN diagnosis occurs in cases of the development of ENB at least six months after the end of radiotherapy; in addition, the radiologic features of ORN involve erosive changes and sequestrations [4]. SCC destroys the jawbone by showing invasive or erosive patterns; the invasive pattern shows cancer penetrating the cortical bone without the interposition of connective tissue; therefore, tumoral islands grow into cancellous spaces [8]. The erosive pattern shows a connective tissue layer separating the cancer from the bone; therefore, the tumor erodes the cortical bone without growing into cancellous spaces [8]. The current case report aims to show the diagnostic challenge of mandibular ENB in a patient with locally aggressive cutaneous squamous cell carcinoma of the lower lip and carrying risk factors for osteoradionecrosis and medication-related osteonecrosis of the jaws.

## 2. Case Presentation

**Figure 1:**
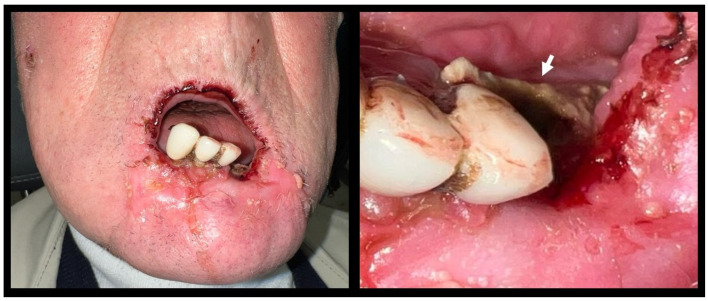
Clinical Examination. On the left: The extraoral examination shows the swollen lower lip due to the locally advanced cutaneous squamous cell carcinoma, incompetent labial seal, vestibular inclination of the lower right incisors and the canine, microstomia, and ulcers of the lower lip. On the right: a detail of the 3 cm area of exposed necrotic bone in the left region of the mandible.

**Figure 2:**
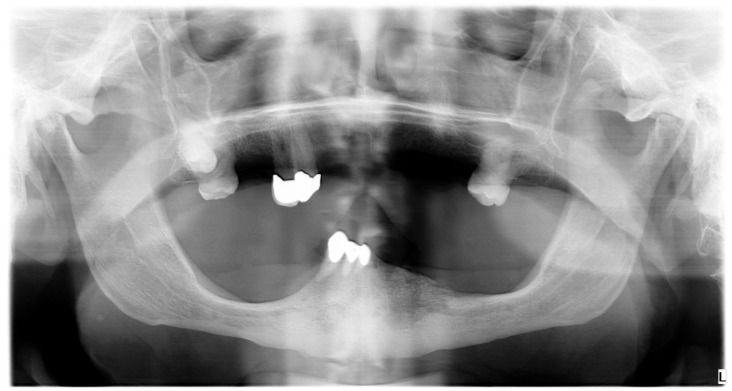
Panoramic Radiogram. The panoramic radiogram shows the osteolytic area overlapping with the lower left mandibular canal.

In March 2023, an 80-year-old ex-farmer male patient came to the Odontostomatology Unit of the “Aldo Moro” University of Bari (Italy) due to feeding difficulty. The cause of the patient’s main complaint was pain in the lower right incisors and the canine during chewing; these teeth showed grade III mobility. The patient showed an incompetent labial seal because of the anterior inclination of these teeth. The patient had microstomia and several ulcers on the lower lip. In addition, the patient showed a painful 3 cm area of ENB in the left region of the mandible (Figure 1 and Figure 2).

**Figure 3:**
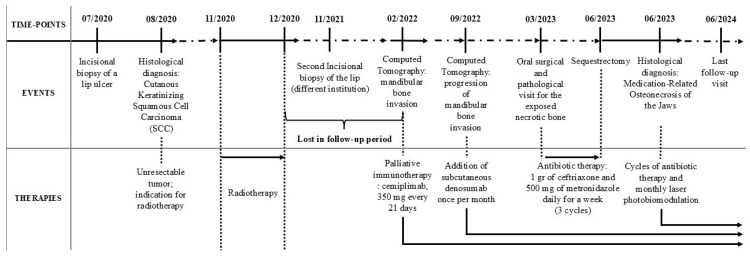
A summary of the patient’s recent medical history. In the middle row, the timeline shows the diagnostic procedures and the events that occurred from the first incisional biopsy of the lip to the last follow-up; in the lower row, the figure shows all therapies the patient received.

The medical history included arterial hypertension, type-2 diabetes mellitus, monoclonal gammopathy, chronic infection by C-hepatitis virus, and post-traumatic epilepsy. In 2007, the patient underwent the excision of a pituitary macroadenoma secreting growth hormone (GH) and causing acromegalia; as a result, the patient developed partial hypopituitarism involving the corticotrope and thyrotrope sectors and secondary hypothyroidism. Figure 3 summarizes the patient’s recent medical history and the events listed in the current report.

**Figure 4:**
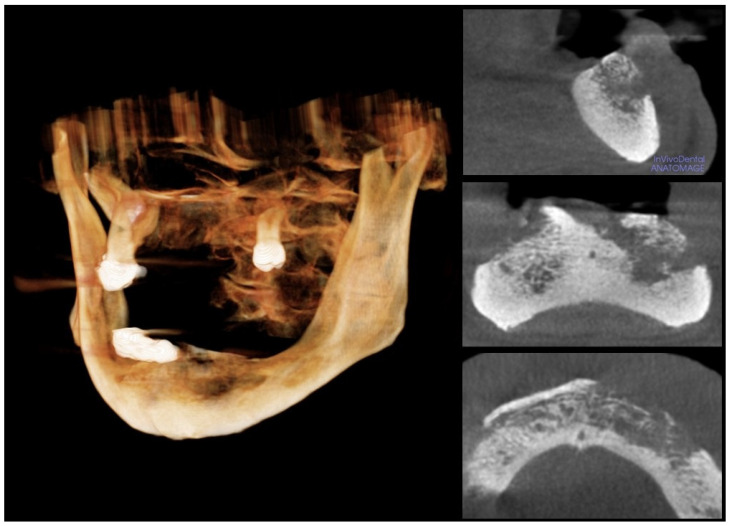
Cone beam computed tomography. The cone beam computed tomography with 3-D reconstruction of the osteolytic area shows the sequestration of the necrotic bone.

In July 2020, the patient underwent an incisional biopsy of a lower labial cutaneous ulcer; the histological examination reported a keratinizing SCC, which developed within actinic cheilitis. In August 2020, the consultant oncologist prescribed radiotherapy because the cancer was unresectable due to the site and the extent of the local invasion; the cycle of radiotherapy lasted from November to December 2020. The patient discontinued oncological follow-up without providing reasons after the end of radiotherapy. In November 2021, the patient received another lip biopsy at a different institution; then, the patient returned to the previous oncologist to reprise the follow-ups. In February 2022, the patient underwent multi-slice computed tomography (CT), which showed the invasion of the subcutaneous tissues of the lower lip and chin; the tumor invaded the external cortical bone of the mandible without signs of osteolysis until it reached the left mandibular angle and the right mental foramen; then, the tumor invaded the terminal tract of the canal of the right mental nerve. The cancer also reached the left mandibular canal by completely infiltrating the homolateral canal of the mental nerve. Therefore, the oncologist prescribed palliative immunotherapy based on cemiplimab, which is an inhibitor of programmed cell-death protein 1 (PD-1) for the targeted treatment of advanced cutaneous SCC; the patient received 350 mg of cemiplimab every 21 days. In September 2022, the CT showed osteolytic activity; thus, the oncologist added the monthly administration of subcutaneous denosumab to the immunotherapy. In March 2023, the latest CT showed no significant changes in the tumor and the patient clinically showed ENB for the first time (Figure 4). Daily, the patient received 15 mg of lansoprazole, 10 mg of ramipril, 100 mg of acetylsalicylic acid, and 150 mg of lamotrigine; the patient also needed 75 mg of levothyroxine to compensate for hypothyroidism, 10 mg of pegvisomant to treat acromegalia, 50 mg of cortisol because of the deficit of adrenocorticotropic hormone, and calcium and vitamin D3 supplementation. Thus, considering such a medical history, the differential diagnosis of ENB involved ORN, stage-III MRONJ caused by a combination of cemiplimab and denosumab, and intraoral extent of the cutaneous SCC.

The first recorded scenario was ORN because the patient underwent radiotherapy in 2020. The oral surgeon formed the hypothesis of stage-III MRONJ because of the combined administration of denosumab, cortisol, and cemiplimab. Eventually, a hypothesis of the cutaneous SCC invading the mandible arose because of the lack of surgical resection of such a malignancy. The aim of the medical team was the histological examination of the ENB after the surgical excision under general anesthesia; thus, the patient underwent a panoramic radiogram, cone beam CT, and a visit by the consultant anesthesiologist. In addition, the patient discontinued denosumab after consulting with the oncologist. The patient received three cycles of antibiotic therapy; each cycle included 1 gr of ceftriaxone and 500 mg of metronidazole daily for a week; 15 days of wash-out occurred between consecutive cycles. However, the patient’s general health conditions worsened due to SARS-CoV-2 infection a few days before the excision, and the oncologist contraindicated the surgery in agreement with the anesthesiologists. Therefore, the oral surgeon performed the sequestrectomy of the ENB and the extractions of the lower teeth under local anesthesia and antibiotic prophylaxis; the histological examination of the ENB confirmed the hypothesis of MRONJ because the sample showed bony structures characterized by wide acellular necrotic regions, large and scalloped Haversian canals, and inflammatory infiltrate; the pathologists found basophilic bacterial colonies and inflammatory infiltrate composed of polymorphonuclear phagocytes, plasma cells, monocytes, and lymphocytes [12]. The wound showed partial healing a month after the surgery. The medical team continues to conduct monthly follow-ups for the patient; each visit includes the delivery of photobiomodulation (PBM) therapy as an adjuvant technique to manage the wound; the PBM protocol involves a diode laser with a wavelength of 810 nm in noncontact continuous wave mode to irradiate 1 W/cm^2^, for a total exposure time of 1200 s per session. The combination of PBM and cycles of antibiotics keeps the MRONJ lesion steady, and the oncological therapy prevents the growth of the SCC. In addition, the authors also delivered PBM to the labial ulcers and have suggested covering these lesions with Vaseline oil to alleviate the pain. Before drafting this manuscript, the last follow-up visit occurred in June 2024; the patient was alive and without symptoms.

## 3. Discussion

ENB may occur as the first sign of several diseases affecting the bone of the jaws and showing overlapping clinical and radiological appearances [2,4,5]. However, the current literature lacks a dedicated flowchart for the differential diagnosis of the primary disease causing the ENB [2,4,5]. As a result of such a challenging differential diagnosis, the treatment of the cause of ENB may be delayed; thus, the QoL of patients becomes worse because of the occurrence of pain, halitosis, dysgeusia, infections, fistulas, trismus, numb chin syndrome, and pathological fractures [1,2,3]. In the current report, the diagnostic hypotheses involved MRONJ, ORN, and local invasion by cutaneous SCC.

### 3.1. Limitations of the Clinical Data in Difficult Differential Diagnosis

The AAOMS guidelines for the diagnosis of MRONJ partially fitted the reported patient because the ENB developed during treatment with antiresorptive therapy and persisted for over eight weeks; however, the history of radiation therapy to the jaws and the invasion of the mandible by the cutaneous SCC represented exclusion criteria for the diagnosis of MRONJ [7]. The patient also partially fitted the diagnostic criteria for ORN because the ENB occurred three years after the end of radiotherapy and failed to heal within the 3 months following the onset of the lesion [4]; however, the patient showed persistence of the invasive cutaneous SCC and the authors could not rule out the presence of such a tumor within the ENB without histological examination, which was necessary to confirm the diagnosis of ORN [2]. The medical history is pivotal for the differential diagnosis of ENB in patients carrying a single risk factor; in 2021, Gaêta-Araujo et al. warned about superimposed predisposing factors, such as patients under anti-resorptive therapy who received radiotherapy in the head and neck region [1]. In the same year, Zadik et al. emphasized the importance of patient-specific radiation dosimetry in the differential diagnosis between ORN and MRONJ; in particular, these authors suggested a modification of the MRONJ diagnostic criteria to include patients who underwent radiotherapy in the head and neck region and received ≤40 Gy in the jaw site where the bone necrosis occurred [13]. In addition, Zadik et al. suggested establishing a new diagnostic category for cases with a disputable origin of the osteonecrosis, namely medication- and radiation-related osteonecrosis of the jaw, as adopted by the guidelines of oral mucositis, which distinguished mucositis caused by radiotherapy, chemotherapy, or combined chemoradiotherapy [13]. In the current case, the authors failed to retrieve data regarding the dose of radiation that the patient received during radiotherapy. Even if the authors could assume that the dose received by the mandible was higher than 40 Gy, as other authors found [13], such an assumption was insufficient to achieve a clear diagnosis of ORN in the study patient. Eventually, the diagnostic challenge was further complicated because the patient developed the ENB during the administration of chemotherapy, and in a period showing evidence of the interruption of the tumoral growth in the CT scans. Cutaneous SCC accounts for up to 20% of all skin malignancies and has an incidence of over 1 million cases per year; most SCCs occur in sun-exposed skin regions [14,15]. The treatment for cutaneous SCC is surgery or radiotherapy in >95% of patients; the remaining <5% show metastatic or locally advanced cutaneous SCC, which is not eligible for surgery and radiotherapy [11]. Such patients have a poor long-term prognosis with a 5-year survival rate ranging from 51% to 64% [11]; in this report, the patient belonged to this category and, thus, received 350 mg of cemiplimab as palliative immunotherapy every 21 days. In 2019, the European Medicines Agency (EMA) approved cemiplimab as the first drug to treat adult patients with metastatic and locally aggressive cutaneous SCC [11]. Cemiplimab restores and enhances the antitumor activity of the immune system against cancers [11,16]. Verkerk et al. administered cemiplimab 350 mg to 151 patients with metastatic and locally aggressive cutaneous SCC every three weeks for 16 weeks [11]. The rate of patients showing an objective response or stabilization of the disease was 54.3% in the 16th week [11]. At the end of the study period, the median progression-free survival was 12.2 months, whereas the median overall survival was 24.2 months [11]. In addition, the authors recorded sixty-eight treatment-related adverse events, which occurred in 29.8% of patients; the most common adverse event was kidney transplant rejection, which occurred in 9.5% of patients [11]. In this report, the early signs of objective stabilization of the disease occurred after the oncologist started the administration of both denosumab and cemiplimab in September 2022; by then, the patient’s disease had stabilized without signs of progression and they did not experience adverse events related to the treatment with cemiplimab. Such findings could suggest that the origin of the patient’s ENB was not the cutaneous SCC; however, such evidence was inconclusive for diagnostic purposes.

### 3.2. Considerations Regarding the Histological Examination of EBN

The current case supports histological examination as the only successful technique to resolve the diagnosis of the disease that causes ENB. In addition to the guidelines for SCC, those for MRONJ and ORN rely mainly on the clinical presentation of these lesions and the patient’s medical history, barely mentioning the importance of histological examination to solve the issues in differential diagnosis [4,7,17]. In 2023, Zisis et al. reported an SCC occurring in an edentulous alveolar ridge resembling a denosumab-induced MRONJ in a 78-year-old osteoporotic woman; based on their experience, the authors stressed that histological examination should be mandatory for all osteolytic lesions due to bisphosphonates or antiresorptive drugs and suspicion for primary or metastatic malignancy should be high [18]. However, several authors agree that histological examination of the exposed bone should not be mandatory for the preoperative diagnosis of MRONJ and ORN; thus, histological examination occurs after surgical resection to exclude the presence of malignant diseases, such as new primary tumors, persisting or recurring tumors, and metastatic disease [5,19].

### 3.3. Considerations Regarding the Targeted Treatment Approach

After obtaining a definitive diagnosis, the authors studied a multimodal treatment in addition to oncological therapy to improve the patient’s QoL, remove the intraoral source of pain, and prevent the worsening of lesions. This therapeutic approach based on the improvement of QoL was in line with the suggestions provided by Yilmaz et al. in 2023 [2]; these authors highlighted the importance of improving QoL as the aim of treatment for oncologic patients carrying BNE, especially in those who survive for a long time. Therefore, first, the patient received antibiotic therapy based on cycles of ceftriaxone and metronidazole as non-surgical treatment for MRONJ [19]; second, the authors delivered PBM to the oral mucosa surrounding the MRONJ site to keep stimulating the healing process; eventually, the patient received wound therapy based on photobiomodulation and protection of the skin for the labial ulcers [20]. PBM is an adjunctive therapy to improve wound healing, relieve pain, and reduce inflammation by applying a laser or light-emitting diode (LED). Depending on the protocols, the frequency of irradiation sessions is 3–8 days; the laser wavelengths range between 588 nm and 1064 nm; the minimum power output is 15 mW and the maximum is 5000 mW; and eventually, the total irradiation time reaches 2400 s per session [20]. In this report, the authors used a diode laser with a wavelength of 810 nm in noncontact continuous wave mode to irradiate 1 W/cm^2^, for a total exposure time of 1200 s per session.

### 3.4. Strengths and Limitations of the Current Case Report

The current report has several strengths, such as showing a complex and multidisciplinary approach to the diagnosis and treatment of ENB and highlighting the weaknesses of the current guidelines for the diagnosis of MRONJ and ORN; in addition, the authors wrote the manuscript according to the latest version of the CAse REport guidelines (CARE checklist guidelines). However, this report suffers from several limitations, such as the lack of information about the period from December 2020 to November 2021. The current manuscript is relevant for clinical practice because it shows viable clinical management for the growing number of oncologic patients carrying superimposed predisposing factors, such as anti-resorptive therapy and radiotherapy in the head and neck region. Future research should improve the differential diagnosis process for ENB in oncologic patients with risk factors for multiple diseases who have developed osteolytic lesions of the jaws.

## 4. Conclusions

The current case report supports the need for histological examination to resolve the diagnostic challenge of he mandibular ENB and to differentiate among osteoradionecrosis, stage-III medication-related osteonecrosis of the jaws, and intraoral localization of cutaneous squamous cell carcinoma.

## Data Availability

The original contributions presented in this study are included in the article. Further inquiries can be directed to the corresponding author.
